# Exploring the microvascular impact of red blood cell transfusion in intensive care unit patients

**DOI:** 10.1186/s13054-019-2572-9

**Published:** 2019-08-30

**Authors:** Geoffroy Hariri, Simon Bourcier, Zora Marjanovic, Jérémie Joffre, Jérémie Lemarié, Jean-Rémi Lavillegrand, Dominique Charue, Thomas Duflot, Naïke Bigé, Jean-Luc Baudel, Eric Maury, Mohamad Mohty, Bertrand Guidet, Jeremy Bellien, Olivier Blanc-Brude, Hafid Ait-Oufella

**Affiliations:** 1 Assistance Publique–Hôpitaux de Paris (AP-HP), Hôpital Saint-Antoine, Service de Réanimation Médicale, 184 rue du Faubourg Saint-Antoine, 75571 Paris Cedex 12, France; 2 Sorbonne Université, Université Pierre-et-Marie Curie-Paris 6, Paris, France; 3 Assistance Publique, Hôpitaux de Paris (AP-HP), Hôpital Saint-Antoine, Service d’hématologie, 75571 Paris Cedex 12, France; 4 Service de Réanimation Médicale, Hôpital Central, Nancy, France; 5 Inserm U970, Centre de Recherche Cardiovasculaire de Paris (PARCC), Paris, France; 6 Normandie University, UNIROUEN, INSERM U1096, FHU REMOD-VHF, 76000 Rouen, France; 7 Laboratory of Pharmacokinetics, Toxicology and Pharmacogenomics, Rouen University Hospital, 76000 Rouen, France; 8 Inserm U1136, F-75012 Paris, France; 9 Department of Pharmacology, Rouen University Hospital, 76000 Rouen, France

**Keywords:** Transfusion, Red blood cells, Endothelium, Microparticles, Vascular reactivity

## Abstract

**Background:**

Red blood cell (RBC) transfusion is a common treatment for hospitalized patients. However, the effects of RBC transfusion on microvascular function remain controversial.

**Methods:**

In a medical ICU in a tertiary teaching hospital, we prospectively included anemic patients requiring RBC transfusion. Skin microvascular reactivity was measured before and 30 min after RBC transfusion. Plasma was collected to analyze intravascular hemolysis and draw the lipidomic and cytokine profiles.

**Results:**

In a cohort of 59 patients, the median age was 66 [55–81] years and SAPS II was 38 [24–48]. After RBC transfusion, endothelium-dependent microvascular reactivity improved in 35 (59%) patients, but worsened in 24 others (41%). Comparing clinical and biological markers revealed that baseline blood leucokyte counts distinguished improving from worsening patients (10.3 [5.7; 19.7] vs. 4.6 [2.1; 7.3] × 10^9^/L; *p* = 0.001) and correlated with variations of microvascular reactivity (*r* = 0.36, *p* = 0.005). Blood platelet count was also higher in improving patients (200 [97; 280] vs 160 [40; 199] × 10^3^/mL, *p* = 0.03) but did not correlate with variations of microvascular reactivity. We observed no intravascular hemolysis (HbCO, heme, bilirubin, LDH), but recorded a significant increase in RBC microparticle levels specific to improving patients after transfusion (292 [108; 531] vs. 53 [34; 99] MP/μL; *p* = 0.03). The improvement in microvascular dilation was positively correlated with RBC microparticle levels (*R* = 0.83, *p* < 0.001) and conversion of arachidonic acid into vasodilating eicosanoids.

**Conclusions:**

Patients displaying an improved microvascular reactivity after RBC transfusion had high blood leukocyte counts, increased RBC microparticle formation, and enhanced metabolism of arachidonic acid into vasodilating lipids. Our data suggested a contribution of recipient leukocytes to the vascular impact of RBC transfusion.

**Electronic supplementary material:**

The online version of this article (10.1186/s13054-019-2572-9) contains supplementary material, which is available to authorized users.

## Introduction

Anemia is a common condition in critically ill patients, due to several cumulative disorders including blood loss, inflammation, and functional iron or vitamin deficiency [1]. Aside from etiology identification and correction, severe anemia (< 7–8 g/dl) requires red blood cell (RBC) transfusion [2]. A recent international survey reported that more than 25% of critically ill patients are transfused during their ICU stay [3]. RBC transfusion may increase oxygen delivery to the tissues, improving the oxygen demand/supply imbalance. Conversely, RBC transfusion could also have harmful effects, including transfusion-related acute lung injury, volume overload, and adverse immunomodulation [4]. In addition, several authors suggested that RBC transfusion may impair endothelial function and consecutively microcirculatory blood flow due to nitric oxide- (NO) and l-arginine-scavenging effects of cell-free plasma hemoglobin and arginase-1 activity, respectively [5]. Transfusion of aged stored RBC into healthy patients and critically ill animal models alters endothelial function, a key regulator of microvascular blood flow [6]. In healthy volunteers, auto-transfusion of aged RBC can alter endothelial-dependent microvascular response to acetylcholine [7], but the effects of non-aged RBC transfusion on endothelial function remain unknown, particularly in injured patients.

We speculated that critical conditions characterized by changes in NO metabolism, inflammatory response, and oxidative balance may modulate the impact of RBC transfusion on endothelial-dependent microvascular reactivity. In this study, we aimed to describe and to analyze the effects on skin microvascular function of transfusing RBC to anemic ICU patients.

## Methods

We conducted a first prospective observational study in an 18-bed ICU in a tertiary teaching hospital. We included adult patients (≥ 18 years) who required RBC transfusion to treat severe anemia (local hemoglobin threshold for transfusion is < 7 g/dl or < 8 g/dl for patients with preexisting cardiovascular disease) after organ failure recovery (decreased of vasopressor dosage < 0.05 μg/kg/min, interruption of sedative drug), in order to limit confusing factors related to support-organ therapy. Exclusion criteria were forearm skin lesions, important soft tissue edema, agitation, recent active bleeding (< 48 h), recent RBC transfusion (< 7 days), repetitive RBC transfusion (> 3), shock requiring high-dose vasopressor infusion (> 0.05 μg/kg/min), and mechanically ventilated patients requiring sedative drugs. Patients could be admitted from the emergency department or medical or hematological wards. In a defined sub-study, we collected blood samples for additional analysis.

Skin microvascular reactivity was measured within 10 min prior to and 30 min after transfusion of one packed RBC unit (leukodepleted, stored in SAG-mannitol solution). The infusion rate of RBC unit, at room temperature, was 2 mL/min during 15 min and was next increased to 4 mL/min. The following parameters were recorded before transfusion: age, gender, comorbidities, mean arterial pressure, heart rate, SAPSII, SOFA score, biological parameters, and packed RBC characteristics (age) were also collected. Citrated blood samples were also collected (21 patients, worsening group *N* = 8, improving group *N* = 13) prior to and after transfusion in order to assess plasma levels of cytokines, cell-free heme, and RBC microparticles and analyze the plasma lipidome.

### Assessment of endothelial function in the skin microcirculation

The endothelial function of the skin microcirculation was measured in the forearm by transdermal iontophoresis of acetylcholine (Ach) [8, 9]. This non-invasive technique allows the local transfer of Ach across the skin, and its vasomotor action on subcutaneous capillaries. Ach solution and a weak electrical current are applied onto the skin, creating local differences in electrical potential and the active migration of ions and molecules bearing a net electrical charge through epithelial layers. The direction and speed of migration can be adjusted using the polarity and magnitude. The total amount of Ach delivered into the skin is related to the current and duration of application (i.e., electrical charge). The iontophoresis drug delivery chamber was attached to the flexor surface of the non-dominant forearm. The negative lead of the current source was attached to the drug delivery chamber, and the positive lead (i.e., reference electrode) to a conductive hydrogel pad fixed onto the wrist. After measurement of baseline blood flow for 60 s, three successive applications of Ach were made, every 60 s, using anodal current (0.12 mA for 12 s each). The drug delivery chamber was loaded with 80 μL of Ach (Miochol®). Variations of blood flow in the skin were assessed by LASER-Doppler Flowmetry technique. A LASER-Doppler Flowmeter probe (Periflux 5000, Perimed), embedded within a heating drug delivery chamber, was used in combination with a current-controlled delivery device (PeriIont, Perimed). LASER-Doppler Flowmeter signals were recorded continuously using an interfaced computer with acquisition software (Perisoft, Perimed). Skin blood flow was recorded for 10 min after Ach iontophoresis was initiated. Skin blood flow was quantified as area under the curve (AUC) (Additional file 1: Figure S1). Based on excellent intra-patient reproducibility previously validated in our hands, AUC changes larger than 10% after RBC transfusion were considered as significant. Skin blood flow analyses were performed by an independent physician who did not participate in patient care.

### Quantification of plasma cell-free heme

Plasma samples were prepared by centrifugation of citrated blood for 15 min at 150*g* and at room temperature (RT). Plasma free from large debris and platelets was prepared by applying 2 additional centrifugations at 2500*g* for 15 min, at RT. Plasma was stored at − 80 °C.

An estimation of extracellular heme levels in blood was obtained by measuring absorbance at 398 nm in plasma, i.e., at the peak of Soret band absorbance [10, 11]. This technique detects heme in all hemoproteins, whether contained within Hb (assumed to be the overwhelming species in blood), or bound to other plasma partners.

### Quantification of plasma red blood cell microparticles

Red blood cell microparticles were identified and quantified in plasma by flow cytometry. Briefly, RBC microparticles were labeled with fluorescent recombinant annexin-A5 (FITC-coupled, Merck) and fluorescent anti-CD235 (APC-coupled anti-glycophorin-A, BD-Pharmingen), analyzed by fluorescence-assisted cell sorting using an LSR-II flow cytometer (BD Biosciences), and compared with size-calibrated microbeads (MegamixPlus Biocytex) and fluorospheres (Flow-Count, Beckman Coulter) to normalize volume (Additional file 1: Figure S2).

### Quantification of plasma arachidonic acid and metabolites

Plasma levels of arachidonic acid (AA) and its metabolites derived from cytochrome P450, 14,15-dihydroxyeicosatrienoic acid (DHET), 11,12-DHET, 8,9-DHET, 14,15-epoxyeicosatrienoic acid (EET), 11,12-EET, 8,9-EET, from lipooxygenases, 5-hydroxyeicosatetraenoic acid (HETE), 12-HETE, and 15-HETE, and produced by its reaction with NO, nitro-AA (NO_2_-AA), were determined by high-performance liquid chromatography coupled to tandem mass spectrometry (HPLC-MS/MS) after protein precipitation, lipids extraction, and saponification as previously described [12] (Additional file 2: Supplemental methods).

### Quantification of plasma cytokines

Eleven cytokines were measured in patient plasma before and after blood transfusion, using Luminex technology (Th1/Th2 Cytokine 11-Plex Human Procartaplex panel, ThermoFischer Scientific): GM-CSF, IFN-γ, IL-1-β, IL-2, IL-4, IL-5, IL-6, IL-12 p70, IL-13, IL-18, and TNFα.

### Statistical analysis

Continuous variables were presented as median and 25th–75th interquartile ranges (IQR). Discrete variables were presented as percentages. Comparisons between groups were made with chi-square test for discrete variables and Mann-Whitney test for continuous variables. To compare changes within one group, the Wilcoxon signed-rank test for paired data was used. All tests were computed with the R software (R Foundation for Statistical Computing). Significance was defined as a two-sided *p* value < 0.05. Correlation matrix for quantified lipids before and after transfusion were built and plotted using R v3.4.1 (R Core Team; 2018), packages Stats (R Core Team; 2018), and Corrplot [13], respectively. Spearman coefficient was used to determine correlations between lipid species, and all *p* values displayed were adjusted using the Benjamini-Hochberg procedure.

The protocol was approved by our institution’s ethical committee—*Comité de Protection des Personnes (CPP Saint-Louis, Paris, France).* This non-invasive observational study did not cause any specific intervention linked to Ach iontophoresis. All patients and their families were informed that anonymous data could be used for academic research and gave their consent.

## Results

### Patients’characteristics and microvascular reactivity

Fifty-nine patients were included; 39% were female (*n* = 23) with a median age of 66 [55–81] years. The main initial reason for ICU admission was respiratory disorder, and 40% of patients suffered from bacterial infection. SAPS II was 38 [24–48] and median hemoglobinemia before transfusion was 6.9 [6.6–7.3] g/dL. Clinical characteristics of included patients are summarized in Table 1.

**Table 1:** Characteristics of included patients according to variations of microvascular reactivity after RBC transfusion. SOFA was recorded at the inclusion, Sepsis-related Organ Failure Assessment. SAPS II was recorded at H24, Simplified Acute Physiology Score. *MAP* mean arterial pressure, *CRT* capillary refill time. Data are expressed as number, percentage or median, and interquartiles (IQRs)

Patients’ characteristics	Worsening (*n* = 24)	Improving (*n* = 35)	*p* value
Women (%)	41	37	0.72
Age (years)	66 [55; 76]	66 [53; 82]	0.91
Body mass index	26 [22; 29]	22 [19; 25]	0.34
SOFA score	4 [1; 5]	3 [1; 5]	0.69
SAPS II	40 [22; 46]	33 [24; 48]	0.77
Length of stay in ICU (days)	9 [4; 16]	11 [4; 22]	0.44
ICU mortality (%)	8	11	0.98
Cause of admission in ICU (%)			
Neurologic	4	11	0.63
Respiratory	29	31	0.85
Circulatory	21	15	0.51
Other	46	43	0.82
Sepsis	33	43	0.46
Comorbidity (%)			
Arterial hypertension	17	35	0.13
Diabetes mellitus	20	2	0.04
Vascular	13	20	0.45
Cirrhosis	4	0	0.22
Biological markers (blood)			
Hemoglobin (g/dL)	7 [6.6; 7.3]	6.9 [6.7; 7.3]	0.53
Hematocrit (%)	21.9 [19.8; 23.2]	21.1 [19.9; 22.5]	0.89
Leukocytes (× 10^9^/L)	4.6 [2.1; 7.3]	10.3 [5.7; 19.7]	0.001
Platelets (10^3^/mm^3^)	162 [40; 199]	200 [97; 280]	0.03
Creatinine (μmol/L)	106 [60; 134]	110 [72;295]	0.56
Proteins (g/L)	62 [55; 67]	61 [53; 69]	0.98
Glucose (mmol/L)	6.7 [6; 7.8]	6.9 [5.7; 8.7]	0.96
Chloride (mmol/L)	105 [98; 105]	102 [98; 106]	0.73
Total bilirubin (μmol/L)	13 [8.2; 26.5]	11 [6; 21]	0.53
Hemodynamic parameters			
MAP (mmHg)	74 [68; 84]	77 [71; 83]	0.88
Norepinephrine (%)	4	11	0.64
Cardiac Index (L/min/s)	2.8 [2.6; 2.9]	3.6 [2.9; 4.3]	0.07
Knee CRT (s)	1.9 [1.5; 2.1]	2.2 [1.9; 3.0]	0.14
Central body temperature (°C)	36.8 [36.3; 37.2]	36.9 [36.7; 37.4]	0.84
Skin temperature (°C)	29.8 [29.0; 30.8]	30 [29.5; 30.8]	0.32
Packed RBC characteristic			
pRBC age (days)	25 [18; 30]	27 [18; 30]	0.34

**Fig. 1:**
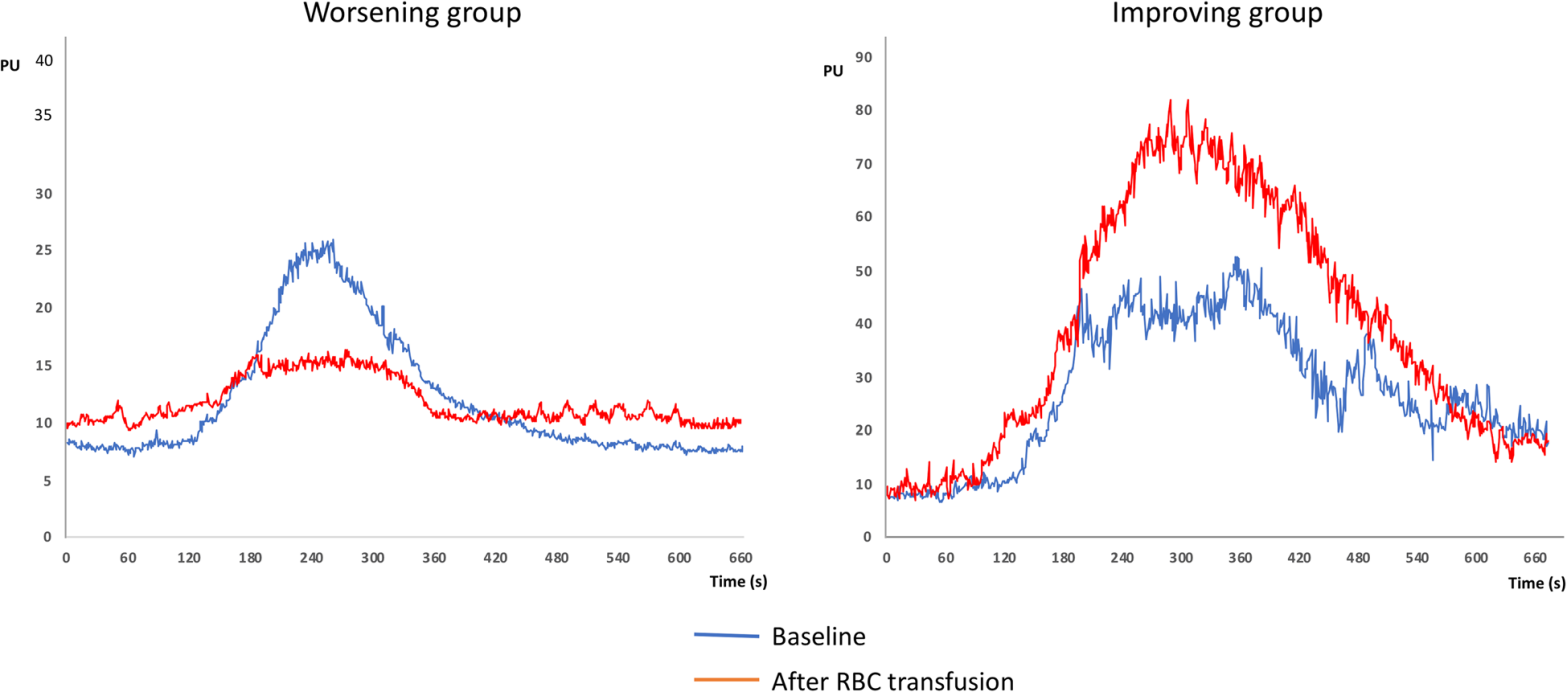
Examples of microcirculatory blood flow measurements in the skin area in 2 patients in response to Ach iontophoresis before (blue curve, H0) and after (red curve, H1). Left, RBC transfusion impaired microvascular reactivity to acetylcholine; right, RBC transfusion improved microvascular reactivity to acetylcholine. PU for perfusion units

Microvascular reactivity was assessed 6 [4–12] days after ICU admission. Acetylcholine iontophoresis was performed 10 min before and 30 min after transfusion of one packed RBC unit. We found that endothelium-dependent microvascular reactivity increased (+ 183 (+ 57; + 459)%), in 35/59 patients after RBC transfusion (improving group), whereas it decreased (− 55 (− 39; − 65) %) in 24/59 patients (worsening group) (Fig. 1). We did not find any significant difference between groups regarding clinical characteristics, comorbidities, and hemodynamic parameters (Table 1). Storage age of the packed RBC units was comparable (25 [18–30] vs. 27 [18–30] days; *p* = 0.34). However, we observed that blood leukocyte count was significantly higher in the improving group compared to the worsening group (10.3 [5.7; 19.7] vs. 4.6 [2.1; 7.3] × 10^9^/L; *p* = 0.001); the difference remained significant for neutrophils, eosinophils, and monocytes populations but not for lymphocytes and basophils (Additional file 1: Figure S3). We also observed higher blood platelet count in the improving group compared to the worsening group but difference between groups was modest (200 [97; 280] vs 160 [40; 199] × 10^3^/mL, *p* = 0.03). Variations of microvascular reactivity after RBC transfusion significantly correlated with baseline blood leukocyte counts (*r* = 0.36, *p* = 0.005) but not with baseline platelet numbers (Additional file 1: Figure S4).

### Plasma cytokine measurement

Among the 11 cytokines tested, none was detectable in the plasma except IFN-γ. Before RBC transfusion, IFN-γ levels were significantly higher in improving group (33.7 [21.5; 56.7] vs. 8.7 [5.5; 20] pg/mL; *p* = 0.01) probably because these patients had higher blood leukocyte count (Additional file 1: Figure S5). However, variations of IFN-γ levels after RBC transfusion were not different between groups (data not shown).

### Plasma markers of intravascular hemolysis

**Fig. 2:**
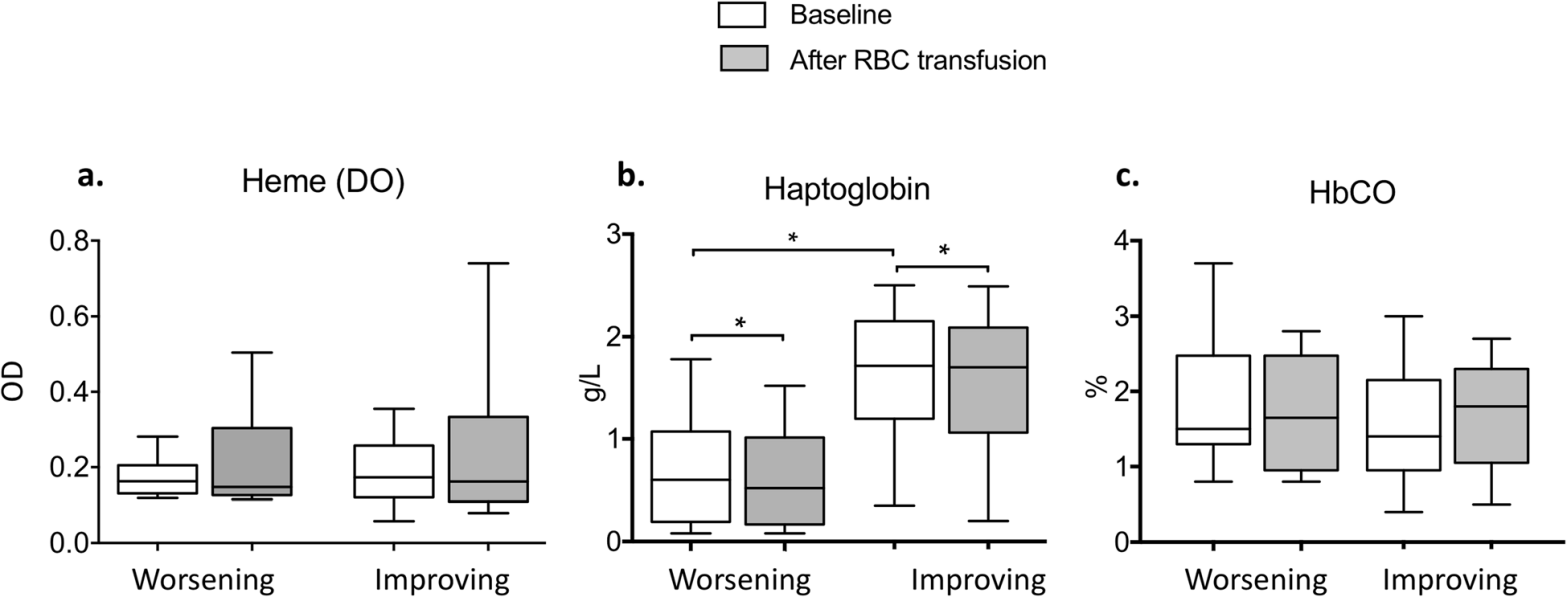
Total heme (**a**), haptoglobin (**b**), and HbCO (**c**) plasma levels before (white) and after RBC transfusion (gray) according to the response to Ach iontophoresis (worsening or improving). OD for optic density

Before RBC transfusion, plasma heme levels were comparable between both groups (0.16 [0.14; 0.18] vs. 0.17 [0.13; 0.25] OD; *p* = 0.60), and concentrations did not change after transfusion. At baseline, haptoglobin level was lower in the worsening group (0.6 [0.2; 1] vs. 1.7 [1.1; 2.1] g/L; *p* = 0.007) and after transfusion, haptoglobin concentration slightly decreased in both groups, but remained detectable for all patients. Finally, another intravascular hemolysis marker, carboxyhemoglobin (HbCO) level, was comparable between both groups before transfusion (1.5 [1.3; 2] vs. 1.4 [1.3; 2]%; *p* = 0.65) and did not change after RBC transfusion (Fig. 2).

### Circulating RBC microparticle quantification

**Fig. 3:**
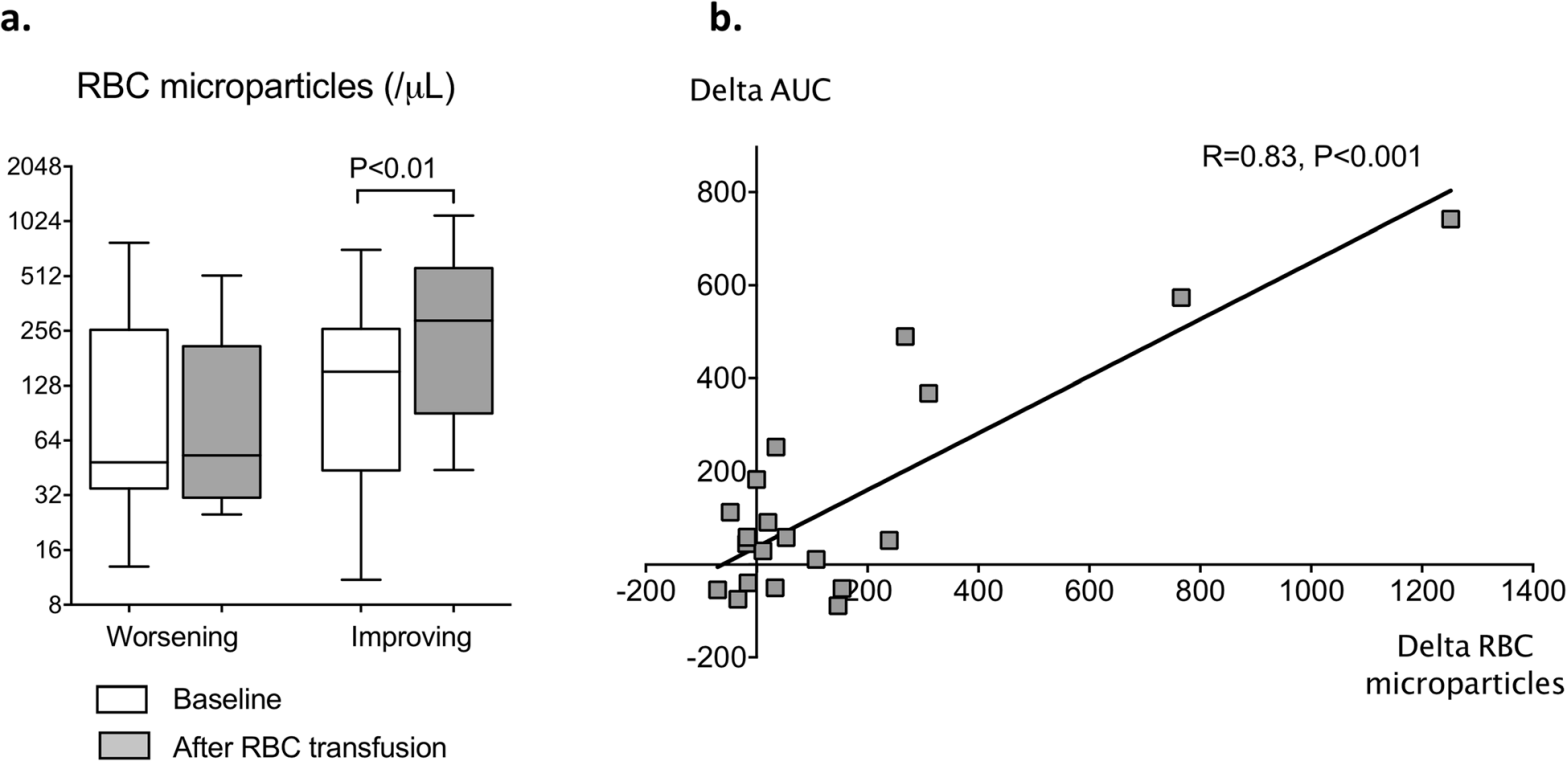
**a** RBC microparticle levels before (white) and after RBC transfusion (gray) according to the response to Ach iontophoresis (worsening or improving). **b** Correlation between variations of RBC microparticles and variations of microvascular response (AUC)

RBC microparticle levels in plasma were comparable in both groups before transfusion (*p* = 0.81), but we found higher RBC microparticle levels in improving patients after transfusion (292 [108; 531] vs. 53 [34; 99] MP/μL; *p* = 0.03) (Fig. 3a). Moreover, we found a positive correlation between RBC microparticles increases and enhanced vasoreactivity in the improving group (*R* = 0.83, *p* < 0.001) (Fig. 3b).

### Plasma lipidomic analysis

**Fig. 4:**
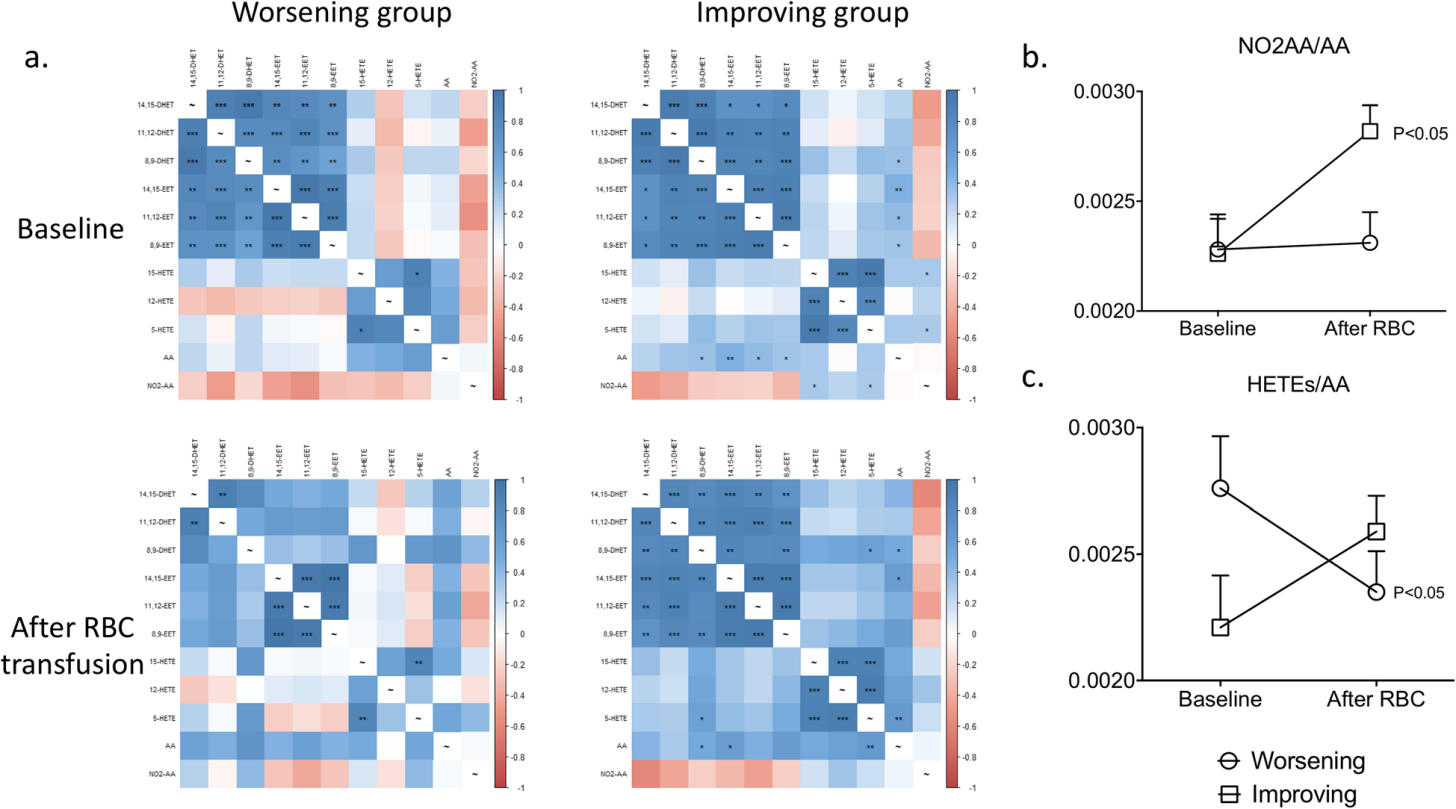
Spearman’s rank correlation matrices for arachidonic acid (AA) and AA metabolites quantified by LC-MS/MS in the plasma of patients before and after transfusion (**a**). Blue and red indicate positive and negative correlations respectively and the color intensity the strength of the correlation. 14,15-DHET, 14,15-dihydroxyeicosatrienoic acid; 11,12-DHET, 11,12-dihydroxyeicosatrienoic acid; 8,9-DHET, 8,9-dihydroxyeicosatrienoic acid; 14,15-EET, 14,15-epoxyeicosatrienoic acid; 11,12-EET, 11,12-epoxyeicosatrienoic acid; 8,9-EET, 8,9-epoxyeicosatrienoic acid; 15-HETE, 15-hydroxyeicosatetraenoic acid; 12-HETE, 12-hydroxyeicosatetraenoic acid; 5-HETE, 5-hydroxyeicosatetraenoic acid; AA, arachidonic acid; NO2-AA, nitroarachidonate. *p < 0.05; **p < 0.01, ***p < 0.001. Given *p* values are adjusted using the Benjamini and Hochberg corrections. NO2AA/AA (**b**) and HETEs/AA (**c**) plasma level ratios before and after transfusion in both groups. HETEs is the sum of 5-HETE, 12-HETE, and 15-HETE

Microparticles being a major source of membrane phospholipids, we investigated the impact of RBC transfusion on lipidomic profile. Before transfusion, LC-MS/MS analysis revealed that correlations between plasma levels of arachidonic acid (AA) metabolites were not different between groups, reflecting a similar balance between AA metabolic pathways (Fig. 4a). However, after transfusion, there was a loss of many correlations in the worsening group but not in the improving group (Fig. 4a). In addition, there was a reduction in plasma levels of AA in the improving group mostly related to increased conversion of AA into NO_2_-AA (Fig. 4b). At the same time, conversion of AA into HETEs increased in the improving group but decreased in the worsening group (Fig. 4c), without change in EETs and DHETs.

## Discussion

In anemic ICU patients, we found that RBC transfusion impacted endothelium-mediated microvascular reactivity, which either improved in a subset of patients, or worsened in others. We observed impaired microvascular reactivity following RBC transfusion in patients with low baseline blood leukocyte count. Non-improving patients fail to remodel their plasma lipidome and to produce RBC microparticles after transfusion.

Fluid infusion, in general, has been shown to damage glycocalyx layer promoting endothelial dysfunction [14]. Here we specifically investigated the effects of RBC transfusion on endothelial-dependent microvascular function, a question still into debate. Previous reports focused on RBC characteristics, few investigated whether host parameters modulate the benefits of RBC transfusion. Risbano et al. [7] found that autotransfusion of 42-day stored (old) RBC in healthy volunteers was deleterious to skin microvascular reactivity, compared to 5-day stored RBC, using Ach iontophoresis. Damiani et al. failed to identify an association of sublingual microcirculatory blood flow [15] with RBC storage time, when comparing fresh (< 10-day storage) with old (> 15-day storage) RBC transfusion in septic patients. Creteur et al. reported beneficial effects of RBC transfusion on sublingual microcirculation, in specific patients with impaired local blood flow at rest [16, 17]. In a meta-analysis of 17 studies, Nielsen et al. [18] reported no correlation between RBC transfusion, tissue oxygenation and microcirculatory blood flow in critically ill patients. RBC storage times and age varied across studies, but the patients could however be segregated into those benefiting and those suffering from RBC transfusion [19, 20]. Our study was performed in anemic, non-severely ill ICU patients after organ failure recovery. There was no relationship between baseline SOFA scores and microvascular outcome (data not shown). For transfusions, we used packed RBC with a median storage time of 27 (22–32) days. There was no correlation between storage times in this narrow range and variations in microvascular reactivity, suggesting that other parameters govern the polarized impact of RBC transfusion on vascular function.

Intravascular hemolysis has consistently been proposed as a key modulator of vascular function after RBC transfusion [21]. Transfusion is largely assumed to induce intravascular hemolysis, to a degree sufficient to compromise NO bioavailability via “NO-scavenging” [5]. Indeed, cell-free hemoglobin combines with NO and degrades into nitrate and methemoglobin. Lysed RBC also releases arginase in plasma, which degrades the NO precursor, l-arginine, thereby decreasing vasodilation and stemming any mobilization of microvascular blood flow. Cell-free hemoglobin, heme, and arginase become acutely relevant during intravascular hemolysis, such as sickle cell disease [22, 23]. Contrasting reports suggested that RBC transfusion might promote vasodilation: Damaged RBC could release ATP, stimulate endothelial eNOS activity, and promote vasorelaxing NO production [24, 25]. Plasma nitrites could also potentially be reduced into NO by cell-free deoxyHb from lysed RBC [26]. However, we observed no increase in classical markers of intravascular hemolysis (CO-Hb, cell-free heme, bilirubin) in our patients after RBC transfusion, besides a negligible (below 10%) drop in haptoglobin levels.

We investigated RBC-derived microparticles [27]. Microparticles (0.1–1.0 μm in diameter) form via outward blebbing and shedding of plasma membrane [28–30] and accumulate with time during packed RBC storage in vitro [31]. RBC microparticles were reported to be vasculotoxic in animal studies and promote NO scavenging via endothelial interactions [30, 32]. However, the storage times of our packed RBC transfused to improving and worsening groups were comparable, arguing against a significant difference in transfused RBC preparations. Moreover, patients with enhanced vasodilation displayed a counter-intuitive, significant increase in RBC microparticle levels, whereas circulating RBC microparticle levels remained low even before and after RBC transfusion in patients displaying deteriorating endothelial-dependent vasodilation. Microparticle levels were strongly, yet unexpectedly correlated with enhanced endothelium-mediated vasodilation, and patients could be segregated according to their generation of circulating RBC microparticles after transfusion. Hence, the leakage of RBC hemoglobin and heme into plasma cannot explain the outcome of RBC transfusion on microvascular reactivity, but the shedding of RBC membrane microvesicles was positively associated to improving vasoreactivity. The mechanisms that drive RBC microparticles generation after transfusion remain unknown.

RBC-transfused patients with improved endothelium-mediated vasodilation displayed previously unknown characteristics: significantly lower baseline platelet and leukocyte counts, particularly in monocyte, neutrophil, and eosinophil populations, compared to patients with worsening vasoreactivity after RBC transfusion. The difference of platelet count between groups was modest, and baseline platelet count did not correlate with variations of microvascular reactivity after RBC transfusion. Conversely, blood leukocyte count was 2.2-fold higher in the improving group and positively correlated with variations of microvascular reactivity after RBC transfusion. We had hypothesized that RBC transfusion could trigger a host inflammatory response, affecting the microvascular reactivity. A cytokine storm release was previously reported in mice after RBC transfusion [33], but these data were not confirmed in human [34]. Of the 11 pro- and anti-inflammatory cytokines that we measured in plasma, most remained below detection thresholds, except interferon-γ. Interferon-γ baseline levels were significantly higher in patients with improving endothelium-mediated vasodilation and might be linked to the blood leukocyte counts. However, interferon-γ levels did not change 30 min after RBC transfusion, when microvascular reactivity had already improved.

It is tempting to speculate about the physiological connections between leukocytes, RBC vesiculation, and microvascular reactivity. For instance, host leukocytes might somehow foster RBC microparticle generation. Richter et al. [35] reported that leuko-reduction decreased RBC content and IL-1α levels in packed RBC units, suggesting that microparticle generation may be induced by released cytokines. Donati et al. described a more favorable effect of leukodepleted RBCs on microcirculatory convective flow when compared to non-leukodepleted RBCs [36]. Once shed, microparticles may feed into pathways that regulate microvascular function. RBC microparticles contain 10-fold more phosphatidic acid and less phosphatidylethanolamine than intact RBC membranes [37]. As a new source of circulating phospholipids, the enzymatic processing of RBC microparticles could generate bioactive fatty acids that facilitate microvascular dilation. In patients with improving endothelium-mediated vasodilation, RBC transfusion strongly remodeled the plasma lipidome, with AA conversion into vasodilating mediators NO_2_-AA and HETEs [38, 39]. In the group with worsening vasodilation, AA metabolic pathways became unbalanced and patients failed to produce these fatty acid derivatives. Moreover, these bioactive derivatives can also incorporate into the endothelial plasma membrane and form a pool releasable upon endothelial activation by shear stress or Ach infusion [40, 41].

We have to acknowledge several limitations. First of all, we performed a pathophysiological study, and our results have to be interpreted with caution. We showed that RBC transfusion impacts on microvascular reactivity, but our small sample size study was not designed to evaluate the effects of transfusion on morbidity or mortality. Skin endothelial measurements have been done after recovery of initial injury, and we cannot exclude different vascular effects of RBC transfusion at early time points. Here, we described the acute impact of RBC transfusion on skin microvascular reactivity, but we did not explore vascular function neither at later stages nor on other organs. Most results came from observational analyses, and we cannot affirm direct causative links between altered microvascular reactivity, leukocyte count, RBC microparticle levels, and plasma lipidome remodeling after RBC transfusion. Several additional factors such as initial disease severity and treatment may modulate the vascular response to RBC transfusion. Finally, RBC microparticle content in stored RBC bags was not quantified.

## Conclusion

We showed that RBC transfusion remodels the microvascular reactivity in ICU patients and microvascular dilation improved in some patients, but worsened in others. We report that circulating leukocyte count positively correlates with microvascular reactivity after transfusion. The transfusion of one packed RBC unit was not associated with significant intravascular hemolysis, but it correlated with increased levels of RBC microparticles and modifications in the plasma lipidome in improving patients only. Larger studies are now required to confirm our results and unravel the clinical significance of microvascular alterations and the putative, vasculo-protective effects of transfusion-induced microparticles.

## Additional files

Additional file 1: **Figure S1.**Typical recording of microvascular skin blood flow recorded by laser doppler flowmetry baseline and following 3 successives iontophoretic applications of Acetylcholine (Healthy volunteer). AUC, area under curve, Ach, Acetylcholine. **Figure S2.** Characterization of RBC microparticles by flow cytometry after gating on small size particules (< 1 μm). **Figure S3.** Baseline leukocyte subset count in the blood according to variation of microvascular reactivity after RCB transfusion. Expressed as median (1IQR-3IQR). *, *P* < 0.05; **, *P* < 0.01. **Figure S4.** A, Correlation between baseline blood leukocyte count and variations of microvascular reactivity after RBC transfusion. B, Correlation between baseline blood platelet count and variations of microvascular reactivity after RBC transfusion.** Figure S5.** Quantification of IFN-γ plasma levels in patients at baseline, just before RBC transfusion. Quantification was performed using Procartaplex method. Expressed as median (1IQR-3IQR). **, *P* < 0.01. (PPTX 409 kb)

Additional file 2: Quantification of plasma arachidonic acid and metabolites. (DOCX 14 kb)

## Data Availability

The datasets used and/or analyzed during the current study are available from the corresponding author on reasonable request.

## Abbreviations

AA: Arachidonic acid
AUC: Area under the curve
Ach: Acetylcholine
ICU: Intensive care unit
IFN: Interferon
LC-MS: Liquid chromatography-mass spectrometry
MAP: Mean arterial pressure
NO: Nitric oxide
RBC: Reb blood cell
SOFA: Sequential organ failure assessment
SAPS II: Simplified acute physiologic score II
