# Supplementary material for: Exploring the microvascular impact of red blood cell transfusion in intensive care unit patients
Source: Crit Care. 2019 Aug 30;23:292. doi: 10.1186/s13054-019-2572-9 (PMC6717366; doi:10.1186/s13054-019-2572-9)
Supplement: Supplementary file 1 — Figure S1.Typical recording of microvascular skin blood flow recorded by laser doppler flowmetry baseline and following 3 successives iontophoretic applications of Acetylcholine (Healthy volunteer). AUC, area under curve, Ach, Acetylcholine. Figure S2. Characterization of RBC microparticles by flow cytometry after gating on small size particules (< 1 μm). Figure S3. Baseline leukocyte subset count in the blood according to variation of microvascular reactivity after RCB transfusion. Expressed as median (1IQR-3IQR). *, P < 0.05; **, P < 0.01. Figure S4. A, Correlation between baseline blood leukocyte count and variations of microvascular reactivity after RBC transfusion. B, Correlation between baseline blood platelet count and variations of microvascular reactivity after RBC transfusion. Figure S5. Quantification of IFN-γ plasma levels in patients at baseline, just before RBC transfusion. Quantification was performed using Procartaplex method. Expressed as median (1IQR-3IQR). **, P < 0.01. (PPTX 409 kb) [file 13054_2019_2572_MOESM1_ESM.pptx]

## Slide 1
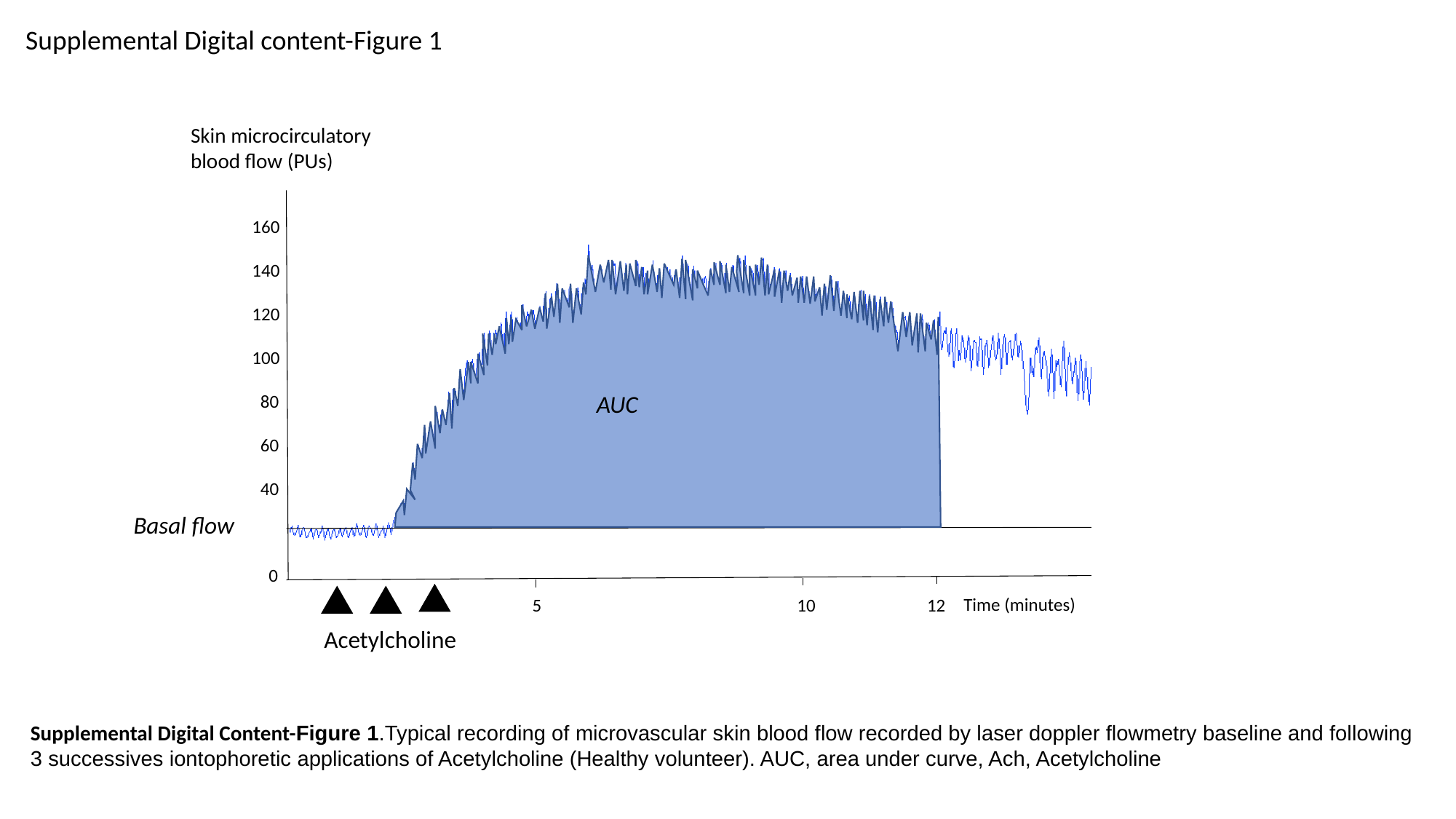

Supplemental Digital content-Figure 1
Skin microcirculatory blood flow (PUs)
160
140
120
100
 80
 60
 40
 20
 0
AUC
Basal flow
Time (minutes)
Time (minutes)
 5		 	10	 12	 	15
Acetylcholine
Supplemental Digital Content-Figure 1.Typical recording of microvascular skin blood flow recorded by laser doppler flowmetry baseline and following 3 successives iontophoretic applications of Acetylcholine (Healthy volunteer). AUC, area under curve, Ach, Acetylcholine

## Slide 2
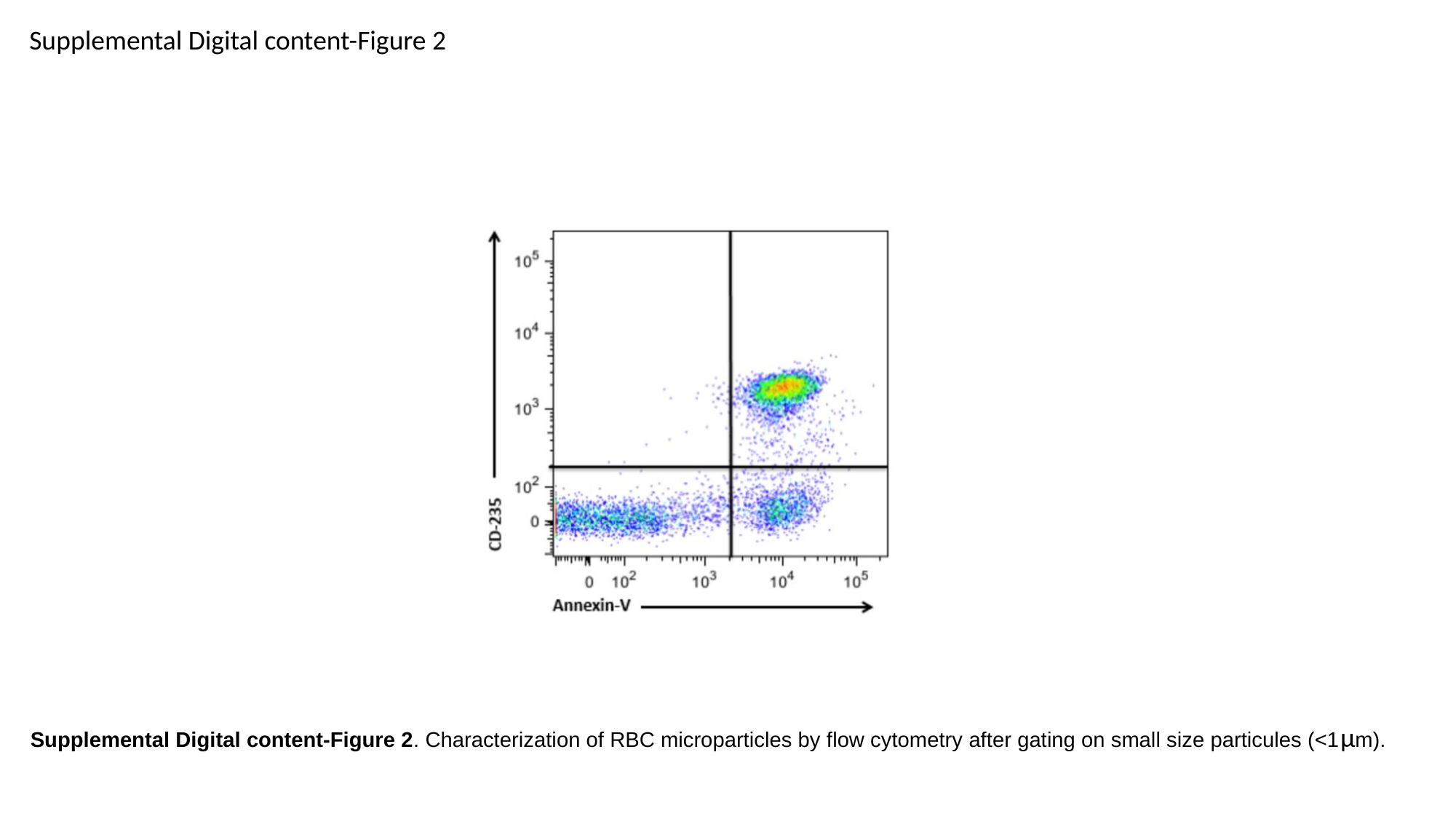

Supplemental Digital content-Figure 2
Supplemental Digital content-Figure 2. Characterization of RBC microparticles by flow cytometry after gating on small size particules (<1μm).

## Slide 3
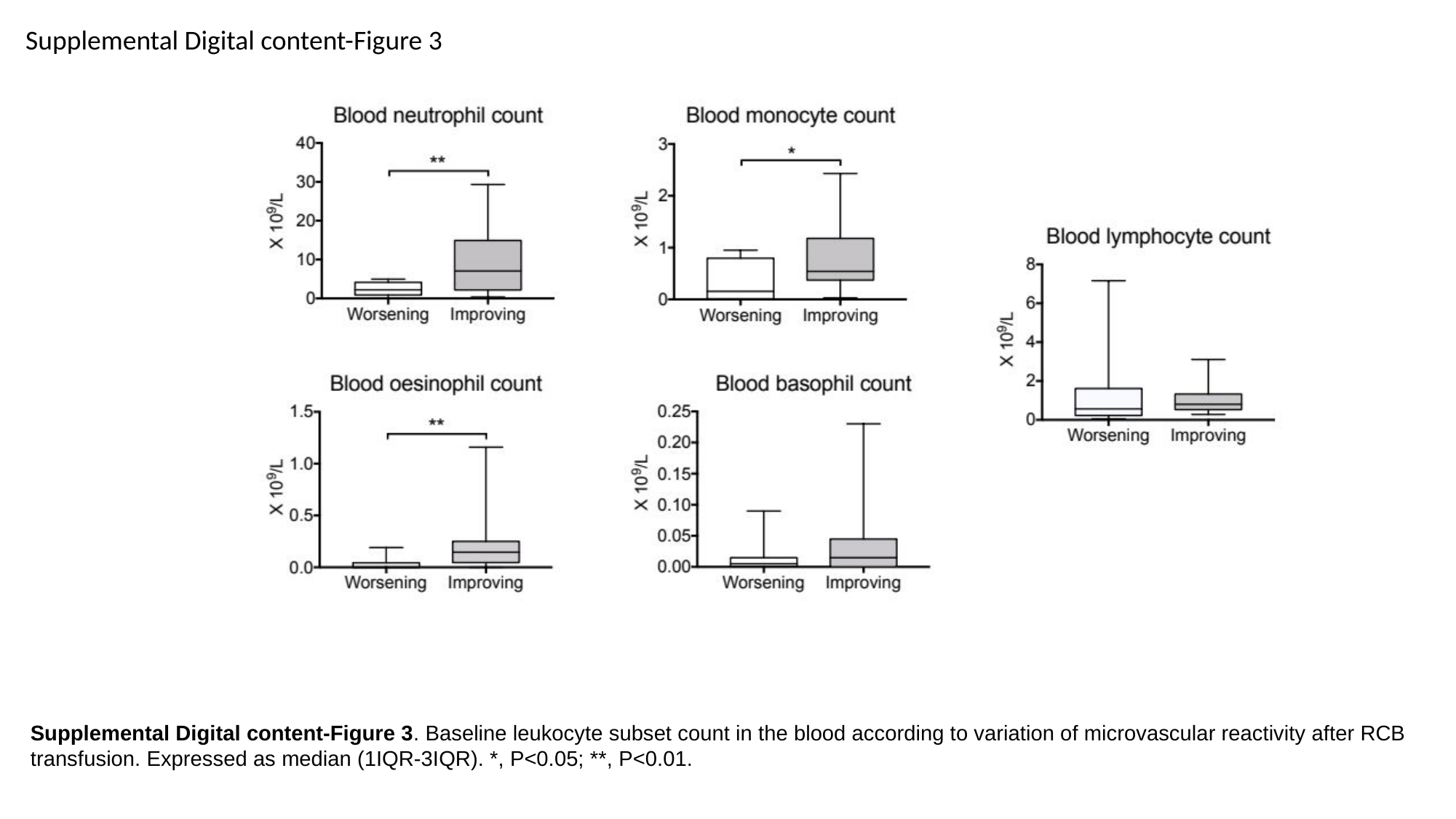

Supplemental Digital content-Figure 3
Supplemental Digital content-Figure 3. Baseline leukocyte subset count in the blood according to variation of microvascular reactivity after RCB transfusion. Expressed as median (1IQR-3IQR). *, P<0.05; **, P<0.01.

## Slide 4
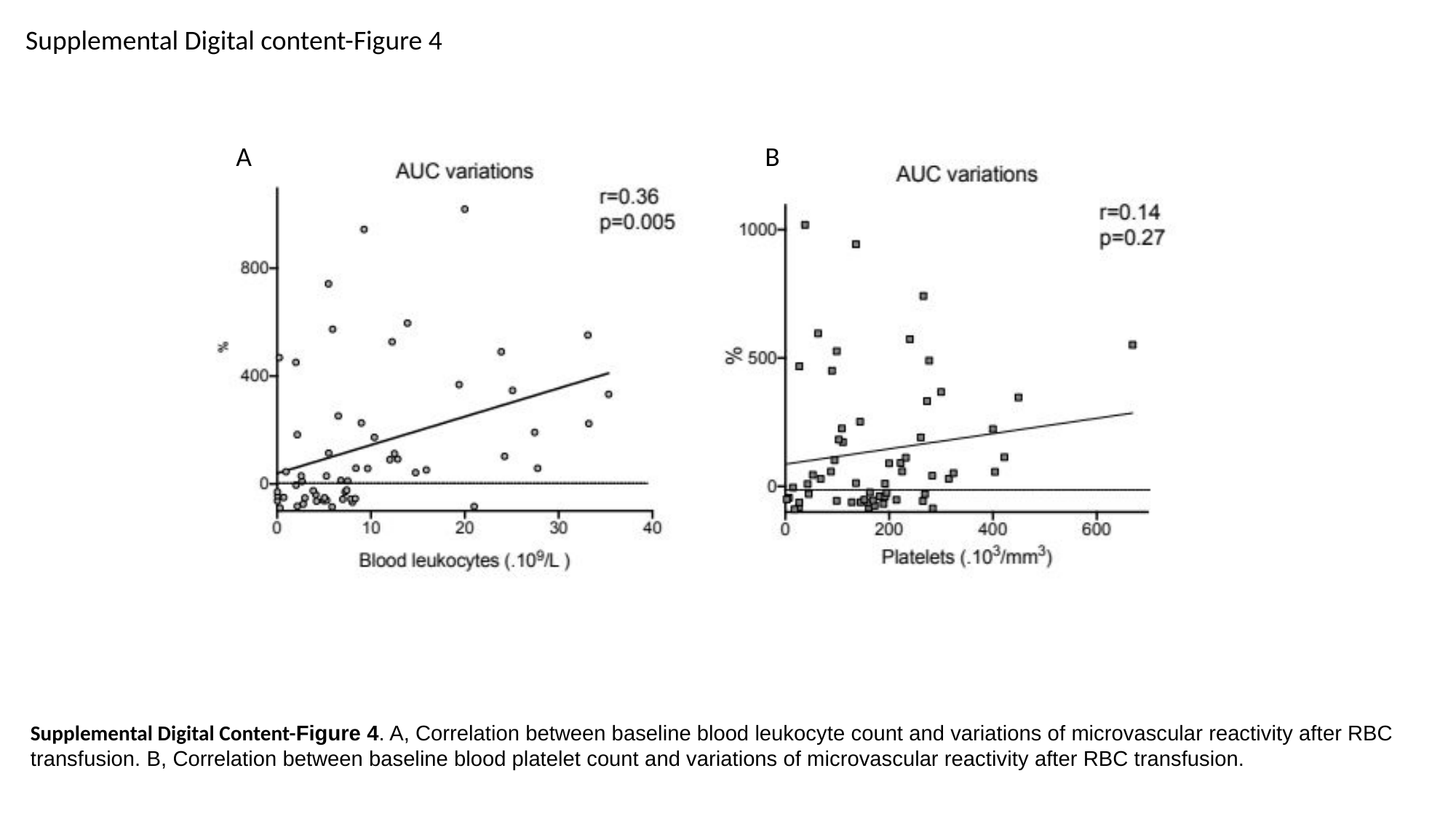

Supplemental Digital content-Figure 4
A
B
Supplemental Digital Content-Figure 4. A, Correlation between baseline blood leukocyte count and variations of microvascular reactivity after RBC transfusion. B, Correlation between baseline blood platelet count and variations of microvascular reactivity after RBC transfusion.

## Slide 5
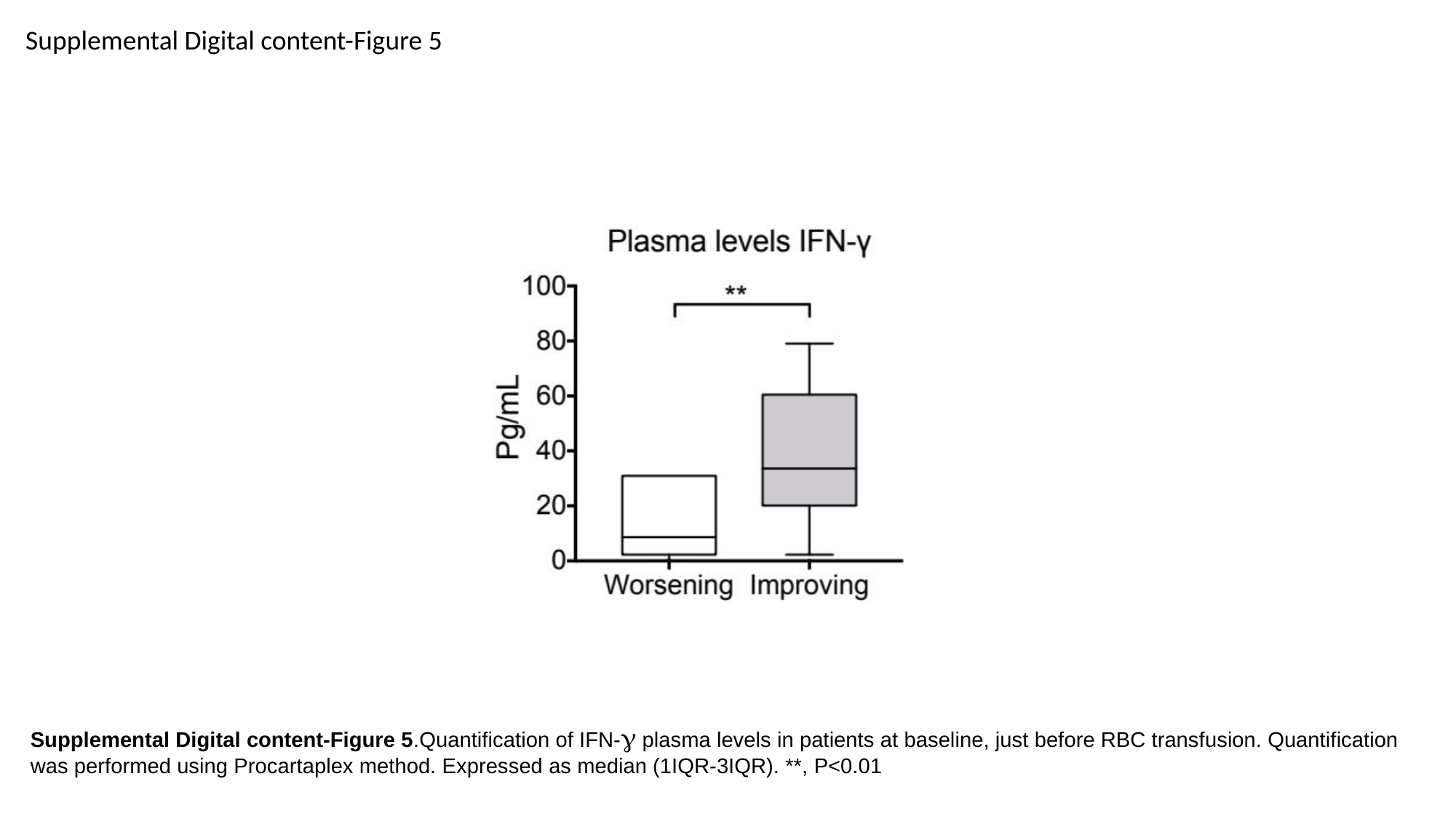

Supplemental Digital content-Figure 5
Supplemental Digital content-Figure 5.Quantification of IFN-γ plasma levels in patients at baseline, just before RBC transfusion. Quantification was performed using Procartaplex method. Expressed as median (1IQR-3IQR). **, P<0.01
