# Supplementary material for: Exploring the microvascular impact of red blood cell transfusion in intensive care unit patients
Source: Crit Care. 2019 Aug 30;23:292. doi: 10.1186/s13054-019-2572-9 (PMC6717366; doi:10.1186/s13054-019-2572-9)
Supplement: Supplementary file 2 — Quantification of plasma arachidonic acid and metabolites. (DOCX 14 kb) [file 13054_2019_2572_MOESM2_ESM.docx]

**Supplemental Digital Content-Supplemental methods**

**Quantification of plasma arachidonic acid and metabolites**

Briefly, 300 µL of plasma were deproteinized with methanol (1:3 vol:vol). After centrifugation, the supernatant was collected, acidified with 100 µL of formic acid 10%, and lipids were extracted by adding 1 mL of dichloromethane. The organic layer was collected and evaporated to dryness under a gentle stream of nitrogen. The resulting crude extract was saponified with sodium hydroxide at +90°C for 20 min to release bound fatty acids into their free form. After cooling on ice, the sample was acidified with formic acid and AA and its metabolites were extracted with 2 mL of dichloromethane. The organic layer was collected, evaporated to dryness, and reconstituted in 100 µL of methanol. Chromatographic separation was performed on a Kinetex C18 column (2.6-μm particle size, 50-mm length × 3-mm inner diameter). The auto-sampler temperature was set at 8 °C, the column oven at 30 °C, the injected volume was 20 μL, and the flow rate was 600 μL/min. The mobile phase was 0.2% formic acid in pure MeOH (solvent A) and 2 mM ammonium formate with 0.2% formic acid in water (solvent B) using the following gradient elution: 0 - 0.5 min, 35% B; 0.5 - 5 min, 35% - 20% B; 5 - 9 min, 20% - 5% B; 9 - 11 min, 5% B; 11 - 13 min, 5% - 35% B and 13 - 15 min, 35% B. MS/MS parameters are given below.

| Analytes | Mass transitions | |  | MS parameters | | |
| --- | --- | --- | --- | --- | --- | --- |
|  | m/z (MS1) | m/z (MS3) |  | DP (V) | CE (eV) | CXP (V) |
| 14,15-DHET | 337,1 | 207,1 |  | -90 | -24 | -9 |
| 11,12-DHET | 337,1 | 167 |  | -80 | -26 | -9 |
| 8,9-DHET | 337,1 | 185 |  | -80 | -22 | -15 |
| 14,15-EET | 319,1 | 219,1 |  | -90 | -16 | -9 |
| 11,12-EET | 319,1 | 207,9 |  | -70 | -16 | -9 |
| 8,9-EET | 319,1 | 154,9 |  | -65 | -16 | -17 |
| 5-HETE | 319,0 | 115 |  | -40 | -20 | -12 |
| 15-HETE | 319,0 | 175 |  | -40 | -19 | -12 |
| 12-HETE | 319,0 | 135 |  | -50 | -19 | -12 |
| NO_2_-AA* | 348,0 | 301 |  | -60 | -25 | -10 |
| AA | 303,0 | 259 |  | -50 | -20 | -10 |

DHET: dihydroxyeicosatrienoic acid; EET: epoxyeicosatrienoic acid; HETE: hydroxyeicosatetraenoic acid; NO_2_-AA: nitroarachidonic acid; AA: arachidonic acid. *Mass transition of NO_2_-AA from Trostchansky et al. (1)

**Reference**

1. Trostchansky A, Souza JM, Ferreira A, Ferrari M, Blanco F, Trujillo M, Castro D, Cerecetto H, Baker PR, O'Donnell VB, Rubbo H. Synthesis, isomer characterization, and anti-inflammatory properties of nitroarachidonate. Biochemistry. 2007; 46:4645-53
